# The maintenance of microbial community in human fecal samples by a cost effective preservation buffer

**DOI:** 10.1038/s41598-021-92869-7

**Published:** 2021-06-29

**Authors:** Chongming Wu, Tianda Chen, Wenyi Xu, Tingting Zhang, Yuwei Pei, Yanan Yang, Fang Zhang, Hao Guo, Qingshi Wang, Li Wang, Bowen Zhao

**Affiliations:** 1 Pharmacology and Toxicology Research Center, Institute of Medicinal Plant Development, Chinese Academy of Medical Sciences & Peking Union Medical College, Beijing, 100193 China; 2 Beijing QuantiHealth Technology Co., Ltd., Beijing, 100070 China

**Keywords:** Metagenomics, Microbiome

## Abstract

In the burgeoning microbiome field, powerful sequencing approaches and accompanied bioanalytical methods have made tremendous contributions to the discoveries of breakthroughs, which favor to unravel the intimate interplay between gut microbiota and human health. The proper preservation of samples before being processed is essential to guarantee the authenticity and reliability of microbiome studies. Hence, the development of preservation methods is extremely important to hold samples eligible for the consequent analysis, especially population cohort-based investigations or those spanning species or geography, which frequently facing difficulties in suppling freezing conditions. Although there are several commercial products available, the exploration of cost-efficient and ready-to-use preservation methods are still in a large demand. Here, we performed shotgun metagenomic sequencing and demonstrated that microbial consortia in human fecal samples were substantially preserved within a temporary storage of 4 h, independent of the storage temperature. We also verified a previous reported self-made preservation buffer (PB buffer) could not only preserve fecal microbiota at room temperature up to 4 weeks but also enable samples to endure a high temperature condition which mimics temperature variations in summer logistics. Moreover, PB buffer exhibited suitability for human saliva as well. Collectively, PB buffer may be a valuable choice to stabilize samples if neither freezing facilities nor liquid nitrogen is available.

## Introduction

Mounting evidence has intensively recognized the pivotal role of gut microbiome in the maintenance of host health and the onset or progression of diseases^1–3^, especially for those investigations on human population, which have provoked great inspirations for health management and therapeutics explorations^4,5^. In the last decade, the rapid development of high-throughput sequencing techniques and powerful analytical methods make considerable contributions to the flourishment of microbiome field via interpreting the massive datasets^6–8^, thereby providing a comprehensive elucidation to the crosstalk between versatile microbiota and host. As the prerequisite, samples are of great significance for the subsequent investigations, such as 16S ribosomal RNA amplicon sequencing, shotgun metagenomic sequencing and metabolomics. Due to the noninvasiveness and convenient acquisition, fecal sample is the commonly used proxy of gut microbiota. Given that the bacterial community in fresh stools could largely retain the real status of gut flora, feces after defecation are recommended for the immediate extraction of microbial DNAs, in order to avoid the contamination of exogenous microorganisms and overgrowth of their own bacteria. Of note, the introduced biases after sampling may substantially alter microbiota consortia, ultimately resulting in a misleading for the following programs.

In practice, freshly collected samples usually go through a freezing step prior to the systematical sequencing and analysis, especially for the large-scale investigations. Immediate cryopreservation at − 80 °C or snap freezing with liquid nitrogen (LN) is considered as the ‘gold standard’ of sample preservation^7,9^. However, microbiome studies in the present era have already intensively expanded in terms of geographic region and species diversity^10–16^, ranging from urban cities (e.g. human, laboratory organisms, companion animals) to remote areas (e.g. population cohorts and wildlife), thus it is infeasible to supply refrigeration facilities or freezing agents. More recently, close attentions have been paid up to the stabilization of sequencing samples, which was overlooked for quite a long time.

Dozens of commercial products are currently available to facilitate the preservation of fecal samples, for instance, OMNIgene GUT kit has been deemed as a better alternative to the ‘gold standard’ by different teams across the world^17–20^, according to assessments of microbial structure and composition. However, the total expenditure on sampling process would be considerable for large-scale studies using OMNI kit (20–25$/kit). Interestingly, other researchers have explored several self-made recipes to evaluate their preservative effects on microbiome, such as ethanol^14^, DMSO-EDTA salt solution (DESS)^21^ and 4% paraformaldehyde solution^22^. Hale et al. found that 100% ethanol could preserve fecal microbial composition to a similar extent to fresh samples of spider monkey^14^. A study involving Japanese adults revealed DESS did not considerably affect the fecal microbiota and OUT profiles^21^. Whereas, coral samples stored with paraformaldehyde solution exhibited apparent variations on microbial structure and composition, relative to liquid nitrogen-frozen coral specimens^22^. Although some recipes displayed beneficial effects, the widespread application of these agents is still impeded due in part to the inconvenience (e.g. the inflammability of ethanol) or lacking of solid and consistence evidence. Therefore, cost-efficient and ready-to-use preservation methods are still in large demand. Of note, Camacho-Sanchez and colleagues once found a nucleic acid preservation buffer (NAP buffer) could stabilize DNA and RNA from rat samples under field conditions up to several months^23^, which has been subsequently applied to preserve sheep fecal microbiome^24^. Given that, NAP buffer may be a valuable preservative in human microbiome studies.

Besides, the storage temperature and duration are another two important factors to affect the performance of microbial DNA. The former mainly includes room temperature and low temperature (including 4 °C, − 20 °C, and − 80 °C). Samples are usually kept at either room temperature or 4 °C for a temporal storage, prior to an ultimate cryopreservation. Storage time distinctly varies from several hours to weeks or months according to schedules of each project, availability of freezing facilities and transportation. Thus, it is meaningful to investigate how preservation time or temperature impact sample stabilities, which bear great missions for the subsequent omics-related studies.

In this study, we conducted shotgun metagenomic sequencing to evaluate the effects of storage temperature (including room temperature, 4 °C, − 20 °C, and − 80 °C) on fecal samples donated by volunteers, in terms of microbial structure and composition. We also verified a self-made preservation buffer (PB) capable of stabilizing fecal samples at room temperature up to 4 weeks and even enduring an extra high temperature condition which mimics temperature fluctuations in summer logistics. Moreover, PB enabled the stabilization of human saliva samples as well, exhibiting suitability to other sample types. Taken together, PB buffer may be a better choice to stabilize sequencing samples when facing the shortage of freezing facilities or logistics constrains.

## Results

### Short-term preservation does not alter fecal microbiota community independently of the storage temperature

**Figure 1:**
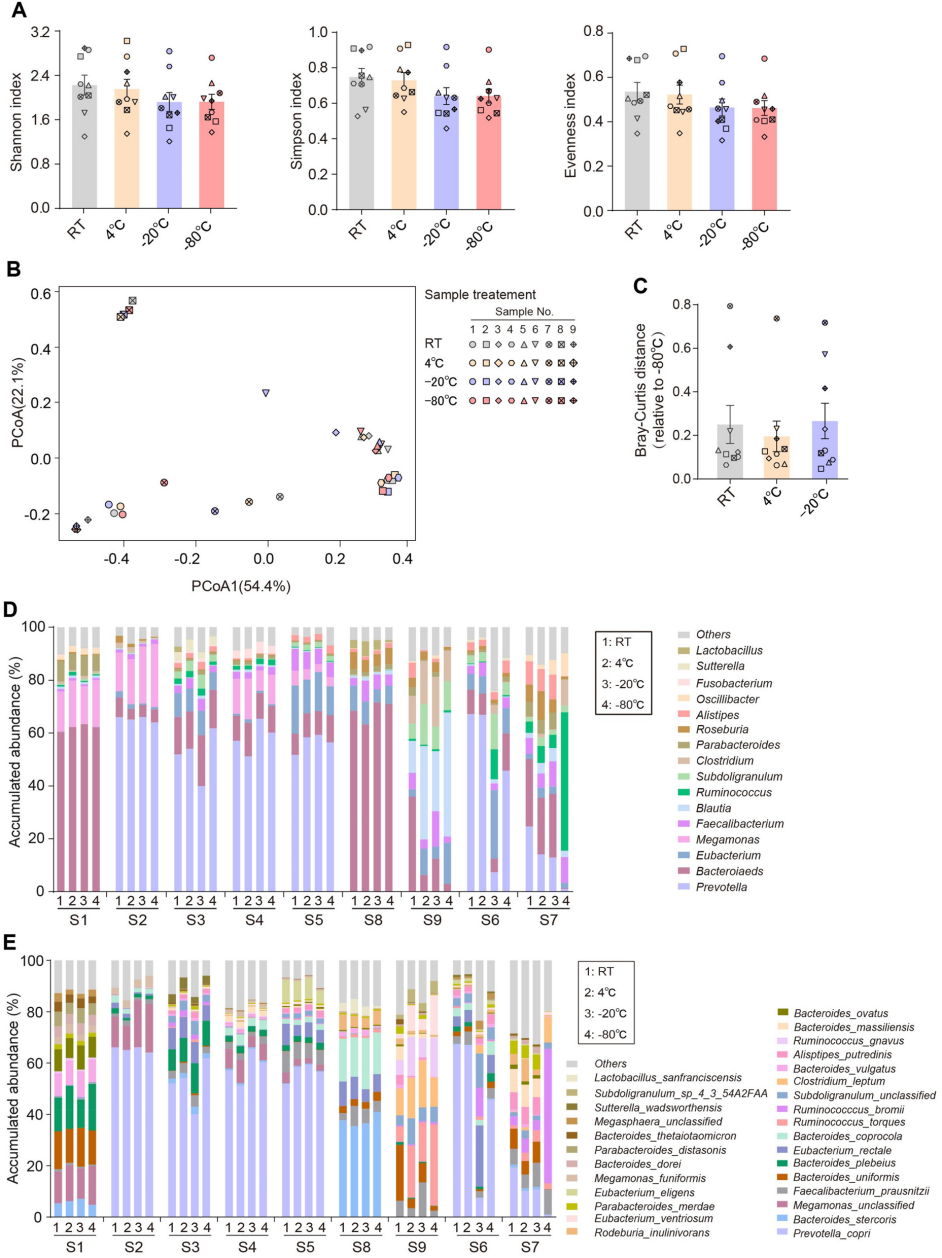
Stability analysis of human fecal microbiota at different storage temperatures within 4 h. (**A**) Shannon, Simpson and Evenness indices of microbial structure in fecal samples under room temperature (RT), 4 °C, − 20 °C and − 80 °C, respectively. (**B**) Principal Coordinate Analysis (PCoA) plots of microbial communities. (**C**) Bray–Curtis distance of microbiome in fecal samples to that in − 80 °C-frozen samples. The relative abundance pattern at the genera (**D**) and species (**E**) level of samples under each indicated condition. N = 9 volunteers/group, each point shape in (**A**–**C**) represents an individual.

Since stool samples commonly experience a temporary storage after sampling, we wondered whether a short period storage would significantly affect microbiome stabilization. Nine volunteers donated fecal samples and each sample was divided into four aliquots to be stored for 4 h at room temperature (RT), 4 °C, − 20 °C and − 80 °C. − 80 °C preservation was used as the control method. Post temporary storage, microbial DNAs were extracted from each aliquot followed by shotgun sequencing. As showed in Fig. 1A, we found a similar α-diversity among all groups estimated by indices of Shannon, Simpson and Evenness, though values were slightly higher in RT and 4 °C groups than freezing groups (− 20 °C and − 80 °C), which was possibly caused by the introduction of experimental microorganisms or the altered growth affected by atmospheric oxygen. These results indicated that the richness and evenness of microbial community in stool samples were not significantly affected by storage temperature under a short-term preservation within 4 h. Principle coordinate analysis (PCoA) further revealed that samples across four different temperature groups tended to overlap each other, exhibiting a high similarity of bacterial structure (Fig. 1B). Analogously, the Bray–Curtis distance of microbial communities to − 80 °C group was not altered among room temperature, 4 °C and − 20 °C groups (Fig. 1C).

As for bacterial composition, we then analyzed the relative abundances of dominant genera and species. At the genera level, six out of nine samples (including samples from No.1 to 5 and No.8) showed an almost identical compositional profile across four temperature conditions (Fig. 1D), suggesting few perturbations caused by different temperatures to microbial community. Other samples displayed a disordered pattern: No. 6 sample had a similar composition under AT, 4 °C and − 80 °C temperatures, the profiles of No. 7 sample at RT, 4 °C and − 20 °C were different from that at − 80 °C, while for No. 9 sample, room temperature storage changed the genera abundance, obviously distinct from other temperatures. These results reflected the presence of inter-individual variations. Clustering analysis clearly exhibited the dispersed patterns of No. 6 and 7 samples (Supplemental Fig. S1). Furthermore, we also observed a consistent species compositional profile with genus, as well as the KEGG functional prediction (Fig. 1E, Supplemental Fig. S2).

Collectively, our results implied that a temporary storage no longer than 4 h could stabilize both microbial structure and composition in human fecal samples, independent of the storage temperature. These findings also provided a hint that the temperature factor did not matter for a temporary preservation (e.g. < 4 h), thus a short-period storage may be a reliable practice to be adopted at the absence of refrigerators and liquid nitrogen.

### A self-prepared preservation buffer (PB) enables to stabilize fecal microbial consortia

**Figure 2:**
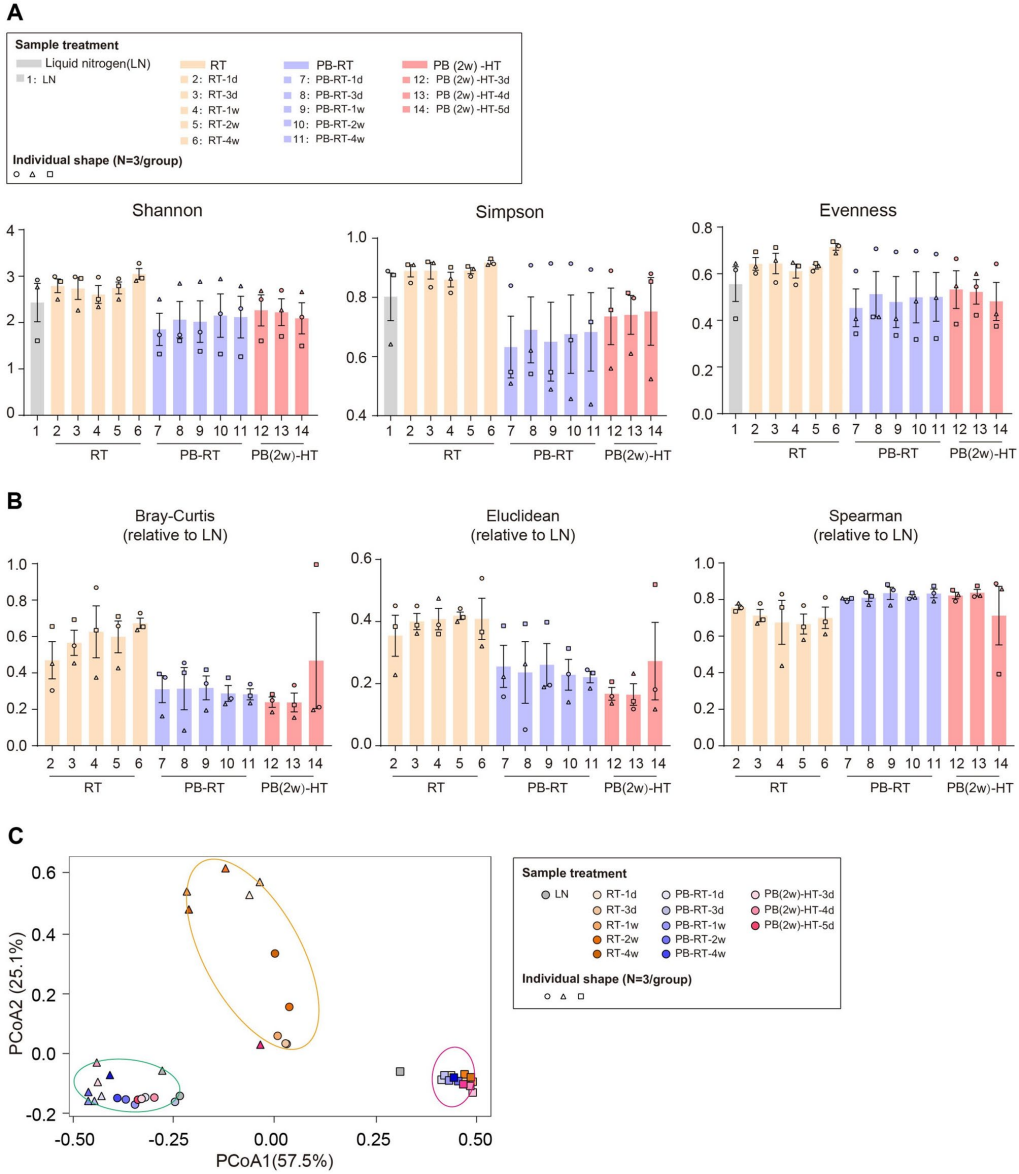
Stability analysis of human fecal microbiota preserved by self-made preservation buffer (PB). (**A**) Shannon, Simpson and Evenness indices of microbiota in fecal samples treated under liquid nitrogen (LN), room temperature (RT), PB buffer, or PB-high temperature preservation. (**B**) Distance and correlation analysis of each group compared with LN group based on Bray Curtis, Euclidean and Spearman coefficient. (**C**) The Principal Coordinate Analysis (PCoA) of fecal microbiome under different conditions, based on the Bray–Curtis dissimilarity index. N = 3 volunteers/group, each point shape represents an individual.

It is widely accepted that the long-time storage at room temperature can destroy the microbial consortia in sequencing samples. In our study, we observed the moderate increase in α-diversity indices of samples stored at room temperature (RT), compared with fecal samples frozen with liquid nitrogen (LN) (Fig. 2A), suggesting the introduction of environmental microorganisms. Specifically, the alterations occurred after even 1-day RT treatment, though the storage period last up to 4 weeks. A nucleic acid preservation buffer (NAP) was previously reported to maintain the quantity and quality of RNA and DNA from mammal samples^23^. We then evaluated whether this lab-prepared preservation buffer (simplified as PB) could be applied to stabilize microbial communities at room temperature or high temperature simulating the temperature fluctuation during summer transportation. For community diversity, PB application at room temperature (PB-RT group) marginally decreased the values of Shannon, Simpson and Evenness indices (Fig. 2A), compared with LN group and the patterns tended to be stable after 3-day preservation. Meanwhile, PB(2w)-HT group samples which underwent 2-week PB preservation followed by an extra 50 °C treatment (lasting for 3/4/5 days) displayed a highly similar profile of bacterial diversity with PB-RT group (Fig. 2A). These results clearly presented the inhibitory role of PB buffer in microbiota shifting under room temperature.

Based on distance analysis (Fig. 2B), the relative distance of AT group to LN group was approximately twice further than that of PB-applied group (for Bray–Curtis, 0.6 vs 0.3; for Euclidean, 0.4 vs 0.2), and the spearman coefficients of two PB groups were higher than RT samples when relative to LN group (Fig. 2B). Moreover, samples stored at room temperature (included in the orange and purple ellipses) drifted heavily from LN-frozen samples (the gray points) (Fig. 2C), while PB-preserved samples (mainly embraced in the green ellipse) tended towards LN samples, exhibiting that PB-treated groups were in close proximity to LN group regarding the microbial structures.

**Figure 3:**
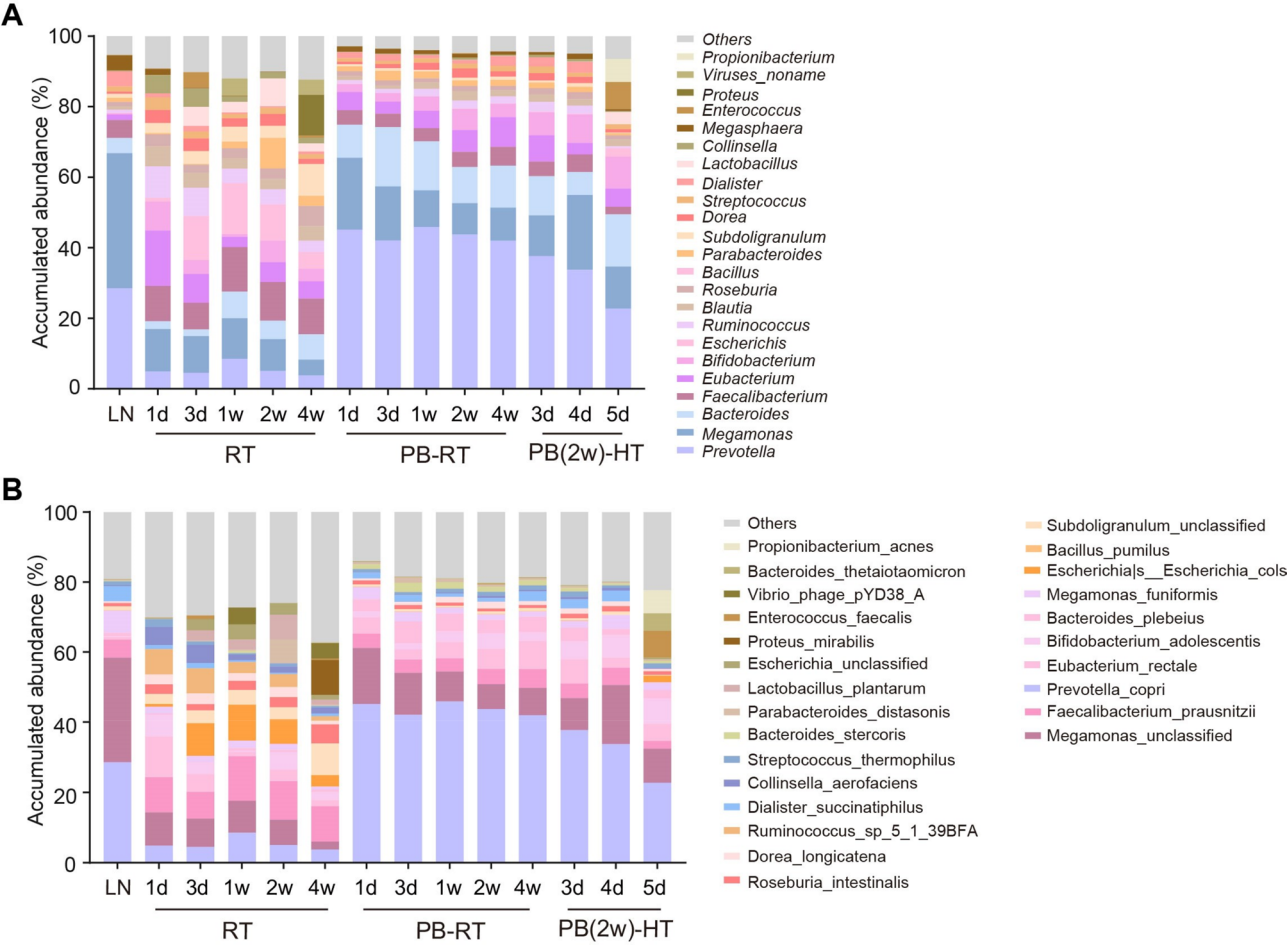
Taxonomic profile of fecal microbiota in human fecal samples stored with PB buffer. The relative abundance profile of microbial communities at the genera (**A**) and species (**B**) level. N = 3 volunteers/group.

Furthermore, we performed compositional analysis at the genera and species levels and found that one-day RT preservation substantially altered genera abundance in microbial community, distinct from the pattern displayed in LN group (Fig. 3A), indicating samples should avoid storage at room temperature, even merely for one day. Conversely, PB maintained the genus composition pattern to a large extent, with some mild perturbations in the proportion of genera, e.g. the increase in *Prevotella*, *Bacteroides* and *Eubacterium*; the reduction in *Megamonas* and *Megasphaera* (Fig. 3A), yet far less than the destruction of composition resulted from room temperature storage. We also observed a great similarity in the relative abundance of dominant genera between PB-RT and PB (2w)-HT groups (Fig. 3A). Consistently, the species composition exhibited an analogous pattern to the observed genera profile (Fig. 3B).

According to these findings, the utilization of self-made PB buffer could facilitate to stabilize microbiome in human fecal samples, promoting samples eligible for the following complicated analysis.

### PB buffer is suitable for human saliva to stabilize microbiota community

**Figure 4:**
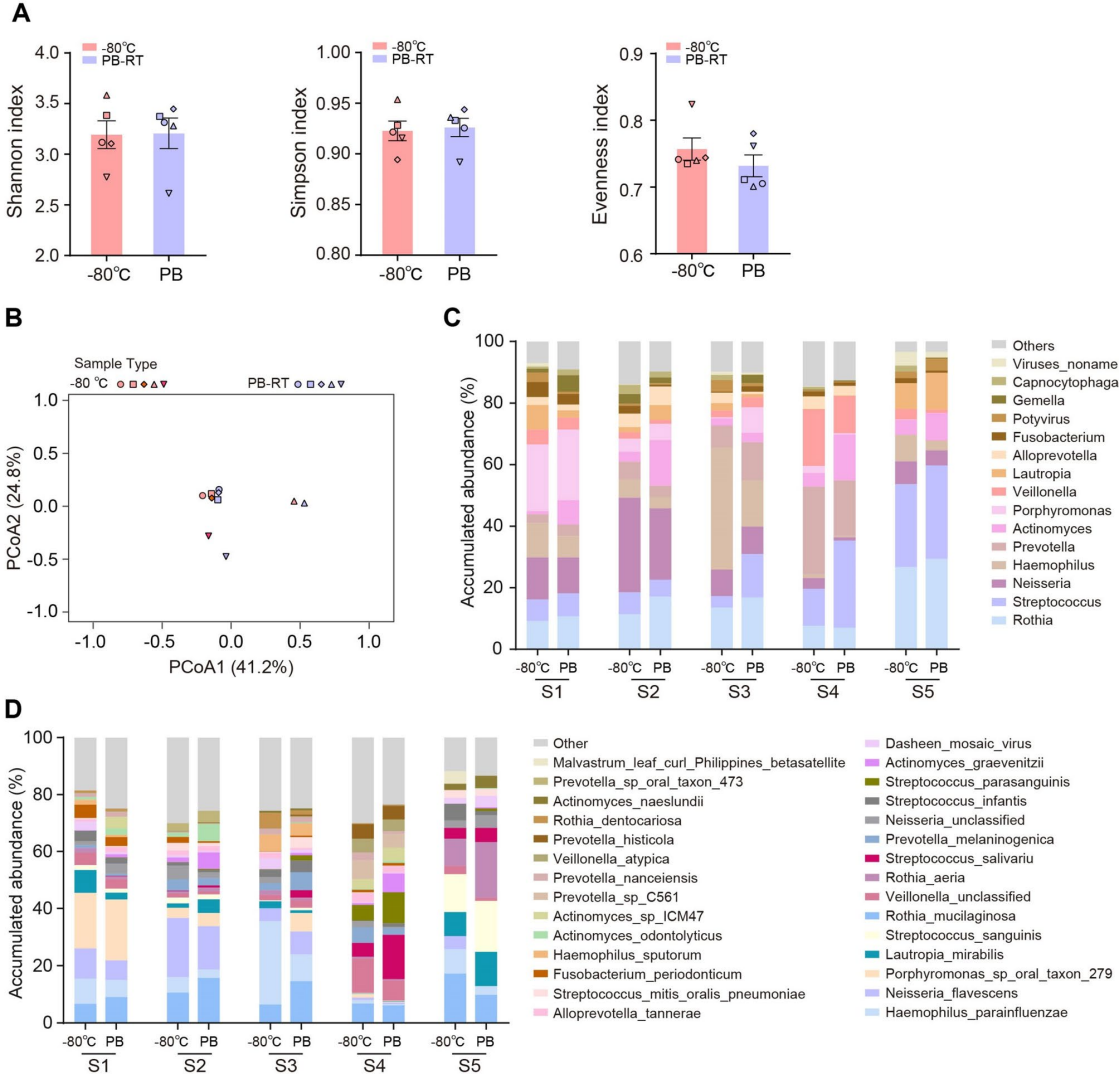
Stability analysis of human saliva samples preserved with PB buffer. (**A**) Shannon, Simpson and Evenness indices of saliva microbiota stored under − 80 °C or room temperature with PB buffer. (**B**) PCoA analysis of microbial communities. Compositional patterns of saliva microbiome at the genera (**C**) and species (**D**) level. N = 5 volunteers/group.

Mammalian microbiota not only vastly colonizes in host gastrointestinal tracts but also resides within or on the body, including lung, oral mucosa, skin and vaginal mucosa^1^, thereby provoking the multifarious samples and the corresponding exploration of preservation methods. In this study, we next tested whether the protective effects of PB would be retained in another sample type. Taking saliva as an example, we collected saliva samples from five volunteers and each sample was divided into 2 aliquots for − 80 °C cryopreservation and PB buffer preservation at room temperature, respectively. According to the aforementioned results of fecal samples preserved with PB buffer (Figs. 2, 3), one-week storage was used as the representative duration in this experiment. Compared with − 80 °C condition, treatment with PB buffer did not change the indices of Shannon, Simpson, and Evenness of microbial community, as depicted in Fig. 4A, indicating the α-diversity of saliva microbiota was stabilized by PB buffer. PCoA analysis also showed that PB-treated samples clustered together with frozen samples (Fig. 4B). Our results consolidated that application of PB buffer could stabilize the structure of saliva microbiome.

For microbial composition, PB group displayed a similar overall profile of dominant genera with that of control samples (Fig. 4C), although there were some alterations such as the relative abundance of *Neisseria* and *Haemophilus* was decreased and *Actinomyces* was increased post PB storage as compared to each − 80 °C control. At the species level, the similar trends could be observed as well (Fig. 4D). Based on the above analysis, we concluded that the self-made PB buffer could prominently preserve the microbial consortia in saliva samples, exhibiting an acceptable suitability.

## Discussion

Researchers have reached a clear consensus regarding the regulatory role of microbiome, particularly for gut microbiota, in human health and diseases based on breakthrough studies in the prevailing field^3–5^. Microbiome-related potential therapies and nutritional interventions have already been promoted such as fecal microbiota transplantation (FMT)^25,26^ and next-generation probiotics^27^. Undoubtedly, the powerful meta-omics techniques and the concomitant analytical methods immensely drive the inspiring findings of the intimate crosstalk between gut microbes and human health^1,6,15^. Recently, accumulating attentions have been paid to a long-neglected fact that samples guarantee the reliability of microbiome studies. In the current work, we found that storage temperatures caused few variations on microbial consortia in human fecal samples in a short-term storage of 4 h, thus providing a relatively safe period for to handle or package samples, without concerning the shifts of microbial community after sampling. Moreover, we demonstrated a cost-effective preservation buffer, PB, could not only keep fecal microbiota stable at room temperature up to several weeks but also enable samples to endure high temperature up to 5 days, as much as the shipping time spent during summer transportation. Also, the PB buffer exhibited suitability for the preservation of human saliva samples. According to our findings, PB buffer may be a promising preservative solution in microbiome investigations, especially for those with a huge sample size or the limited budget.

Preservation methods naturally came out as the storage demand was raised. Researchers usually evaluate the effects of various methods on microbial community via 16S ribosomal RNA sequencing, which is a popular tool to interpret the complex interplay between microbiome and host. This technology provides a comprehensive understanding of the microbial structure, taxonomic composition, yet several limitations apparently exist, for instance, the limited taxonomy resolution down to species level^28^. Our another work also confirmed that 16S sequencing provided less consistent data with whole-genome shotgun sequencing (WGSS), as well as the inaccurate functional predication (unpublished data). As a result, the mere focus on microbial structure may not thoroughly mirror the stabilization of microorganisms in sequencing samples. Due to the prosperous development of meta-omics, several groups have recently applied meta-proteomics or metabolomics individually or in combination with 16S sequencing to examine the effects of preservation methods on microbiome^20,29^. Herein, we performed the more advanced technology, WGSS, instead of 16S rRNA amplicon sequencing, to assess each preservation factor. Besides the measurement of α-diversity parameters, our work provide more in-depth information, including comparative analysis of the relative abundance patterns of dominant genera and species, as well as the predicted KEGG functional profiles.

Nowadays, two major types of preservation methods are available to store sequencing samples, including commercial items and self-prepared buffers. To our knowledge, there are approximately a dozen commercial products with certain reputation such as OMNIgene GUT, Norgen, RNAlater, Shield, MGIEasy, Longsee, fecal occult blood test (FOBT) cards, and fecal immunochemical test (FIT) tubes^17,18,30,31^. Among them, OMNIgene GUT kit displays a better performance to stabilize stool samples as multiple teams have reported^18,20^. Interestingly, RNAlater did not constantly inhibit the shift in microbial community after sampling, unable to sustain its well-known role in RNA protection^17,18^. Two medical tools (FOBT and FIT) were also indicated as acceptable choices for fecal sample collection and storage in future sequencing^31^. Nonetheless, the total cost on the initial sampling step would be prohibitive for large cohorts when using commercial products. Taking OMNIgene kit as an example, it will cost 2000–2500$ for a project with 100 samples in light of the price of 20–25$ per kit, not to mention the vast expenditure on the subsequent sequencing and analysis. Therefore, the application of cost-efficient preservatives with good performance will reduce sampling cost a lot. Ethanol has been mentioned in previous studies regarding its impacts on microbial profiles in fecal samples^30–32^. Specifically, 100% ethanol preserved microbiome structure better than 70% ethanol^16^ and 95% ethanol^31^, as compared to − 80 °C cryopreservation. Unfortunately, the universal application of ethanol does not come as the reagent shows up in the list of dangerous goods, thereby leading to the restricted transportation. Other teams once revealed DMSO-related preservation buffers exhibited few interruptions to α- and β-diversity of gut microbiota in the feces of Japanese adults^21^, even performed as efficiently as snap freezing did in coral specimens^22^. However, elaborate experiments are still needed to confirm the beneficial effects, such as the evaluation of storage period and temperature.

Aiming to find a time-saving and cost-efficient preservative, we noticed that Camacho-Sanchez et al. reported a self-made nucleic acid preservation (NAP) buffer could protect RNA and DNA from mammal samples under field conditions by assessing the quantity and quality of nucleic acids extracted from rat (*Rattus rattus*) samples^23^. Subsequently, this buffer was employed by Menke et al. to evaluate the whether it could preserve the microbial community of wildlife^24^. In their study, researchers took sheep (*Ovis aries* sp.) stools as experimental objects and assessed the preservative effects of NAP buffer and two DNA/RNA preservative media, RNAlater and DNA/RNA Shield, compared with immediate-freezing treatment. For the 10-day storage period, NAP buffer had a better performance to prevent the shift of sheep fecal microbiota after sampling^24^. This interesting work implied the potential application of NAP buffer in sample preservation for microbiome research. Given that, we wondered whether the NAP buffer (namely PB buffer in the context) could stabilize microbial community in human samples. To thoroughly assess it’s efficacy, we introduced shotgun sequencing and more in-depth analysis (e.g. compositional profile down to genus and species level, functional profiling), not 16S sequencing and diversity evaluation as exhibited in previous work. In this study, we concluded that PB buffer could be a valuable alternative to the ‘gold standard’ of sample handling, when facing difficulties in suppling freezing equipment and liquid nitrogen. We draw this conclusion basically based on the following three reasons: (1) Longer preservation period. PB buffer could stabilize fecal microbiota up to 4 weeks at room temperature, with no significant shift in community structure, as well as the genera and species composition, as compared to flash freezing samples. The storage duration of 4 weeks in our work is obviously longer than most of preservation methods (e.g. 4–10 days for RNAlater^24,31^, FOBT^31^ and stool collection tubes^19^) or agents (e.g. 2–8 days for ethanol^14,32^, 3 weeks for DMSO-related solution^21^) have reported. (2) Acceptance to high temperature. PB buffer favors fecal samples to endure a high temperature condition mimicking temperature variations in summer logistics. (3) Low cost. PB buffer is readily available and cost-efficient as the used reagents are common chemicals, thus researchers can greatly cut down sampling expense and focus on the following sequencing. As mentioned above, it will cost thousands of dollars to purchasing commercial products for a project including 100 samples, while the expense of PB buffer can be negligible. Nevertheless, we still need to verify the protective roles of PB buffer across both sample types and species for an abroad promotion, though the human saliva microbiome was not significantly affected by PB buffer treatment. Additionally, a large population is quite necessary to provide solid evidence for the verification work, in light of the obvious inter-individual variances among volunteers. Furthermore, other powerful approaches and analytical methods may facilitate the assessment and discovery of effective preservatives, which could involve in the future research planning.

In summary, we demonstrated that the human fecal microbiota was not perturbed in a temporary storage, no matter which storage temperature was chose, including room temperature, 4 °C, − 20 °C, and − 80 °C. For a long-term preservation, a lab-prepared preservation buffer (PB) could properly maintain microbial profiles for up to four weeks at room temperature, and even sustain an extra five high temperature days. Besides, PB was also favorable to protect the microbial community in human saliva. Therefore, our work provided a suitable alternative to immediate freezing for the subsequent fecal microbiome analysis, particularly under conditions where refrigeration and cold chain transportation are not feasible.

## Material and methods

### Sample collection and storage

Freshly collected feces and saliva samples were immediately divided into aliquots according to the experiment design. All aliquots were then performed under each indicated conditions.

1. For evaluation of storage temperature in a short-term (4 h) preservation, nine volunteers donated the fresh stool samples and each sample was divided into 4 parts for the following conditions: storage at room temperature (RT), refrigerating at 4 °C, immediate freezing at − 20 °C or − 80 °C.
2. For the usage of preservation buffer (PB), fecal samples were contributed by 3 participants and each sample was split into 14 aliquots, belonging to the following groups: the liquid nitrogen (LN)-treated group, the RT group (preserved for 1 day, 3 days, 1, 2 and 4 weeks), the PB-RT group (underwent with the same duration as RT group), the PB (2w)-high temperature group (including a pre-storage with PB buffer for 2 weeks followed by an extra 50 °C preservation for 3/4/5 days).
3. For the assessment of suitability, each sample from 5 volunteers was divided into 2 parts for the cryopreservation at − 80 °C and the application of PB buffer at room temperature for one week.

As for the preservative, the recipe of self-made preservation buffer (PB) was consisted of 20 mM ethylenediaminetetraacetic acid (EDTA) disodium salt dihydrate, 25 mM sodium citrate trisodium salt dihydrate, 5.3 M ammonium sulfate, referring to the previous reported NAP buffer with some mild modifications^23^. 2 ml PB buffer was used for each treatment to sample aliquot (~ 0.5 g feces or 2 ml saliva).

### DNA extraction

The microbial genomic DNA of human stool samples (~ 200 mg/treatment) and saliva samples (~ 2 ml/treatment) were extracted using DNeasy PowerSoil kit (Qiagen) according to the manufacturer’s instructions. For feces preserved with PB buffer, before the extraction process, samples were washed with 1 ml sterilized PBS (Na_2_HPO_4_ 8 mM, NaCl 136 mM, KH_2_PO_4_ 2 mM, 2.6 mM KCl; Solarbio) and vortexed. After centrifugation at 12,000 g for 3 min, the supernatant was discarded to remove PB buffer and the pellets were saved for the following DNA extraction. For human saliva, the wash step was skipped to retain microbiota as much as possible. Samples treated with PB buffer or not were centrifuged at 12,000×*g* for 3 min to separate the supernatant, then the remained pellets were subject to the same subsequent process as feces samples. The extracted DNA was evaluated by 1% agarose gel electrophoresis. DNA concentration and purity were determined with NanoDrop 2000 UV–Vis spectrophotometer (Thermo Fisher Scientific) and Qubit 3.0 fluorometer (Thermo Fisher Scientific).

### Shotgun sequencing

Library preparation for shotgun sequencing was performed using the KAPA HyperPlus Library Preparation kit (KAPA Biosystems) for fragmentation of input DNA following the manufacturer’s instructions. The libraries were quantified by using KAPA Library Quantification Kits (KAPA Biosystems) following the manufacturer’s instructions. Libraries were constructed with an insert size of approximately 350 bp, followed by high-throughput sequencing to obtain paired-end reads with 150 bp in the forward and afterward directions. Shotgun sequencing was performed on an Illumina NovaSeq 6000 System (Illumina). Cluster generation, template hybridization, isothermal amplification, linearization, blocking, denaturing and hybridization of the sequencing primers were performed according to the workflow indicated by Illumina.

### Quality control of shotgun sequencing data

Low quality reads were removed from the raw data by using MOCAT2^33^. Sequencing adapters were removed by using Cutadapt software (version v1.14,-m 30). Then SolexaQA package was used to remove the reads with threshold of less than 20 or the length of less than 30 bp. The reads which could be aligned with the human genome (H. sapiens, UCSC hg19) were cleaned by using SOAP aligner software (v2.21, -M 4 -l 30 -v 10)^34^, and the rest reads were used for further analysis. The detailed information regarding reads were provided as Supplemental Table S1.

### Data analysis

(1) The alpha and beta diversities were calculated by Mothur1.30.2, based on the taxonomic information. The alpha diversity was assessed by the indices of Shannon, Simpson and Evenness. The beta diversity was assessed by Bray–Curtis distances and Eluclidean distances. (2) The principal coordinate analysis (PCoA) was calculated based on the taxonomy information. (3) Microbial community composition was analyzed using Metaphlan2 software. Briefly, the query reads were mapped against the reference genomes in RefSeq database (version 82) with a 97% identity threshold. The reads that mapped to a single reference genome was labeled with the NCBI taxonomic annotation. The reads that matched multiple reference genomes were indicated by the last common ancestor (LCA) of each label according to the NCBI taxonomy. (4) Functional analysis was performed using the HUMAnN software with the default parameters to generate results of KEGG levels. Specifically, RefSeq-derived genes from directly observed exhaustive gapped alignments were annotated according to KEGG Orthology group (KO)^35–37^. To improve the KO profile accuracy for low-abundance genes, the KO profiles were separately predicted from reference genomes and the predicted profiles were used to augment the estimates of low-abundance KOs as previously reported^38,39^.

### Statistical analysis

Data in column charts were showed as means ± SEM (Standard Error of Mean). GraphPad Prism was used for the statistical analysis. The significance among groups was assessed by one-way ANOVA followed by Newman-Keuls post hoc tests. *p* < 0.05 was considered statistically significant.

### Ethics approval and consent to participate

The volunteers contributing stool and saliva samples were recruited as a part of research protocol number 2020LL-3 approved by the Ethics Committee of Third Affiliated Hospital of Qiqihar Medical University, and written informed consent was obtained from each volunteer. All experiments in the study were performed in accordance with the Helsinki Declaration.

## Supplementary Information

Supplementary Information.

## Data Availability

The raw data of experiments in this study acquired by shotgun sequencing have been deposited in the NCBI Sequence Read Archive (SRA) database (BIOProject: PRJNA730593, Accession number: SRP320213).
