# Supplementary material for: The maintenance of microbial community in human fecal samples by a cost effective preservation buffer
Source: Sci Rep. 2021 Jun 29;11:13453. doi: 10.1038/s41598-021-92869-7 (PMC8242035; doi:10.1038/s41598-021-92869-7)
Supplement: Supplementary file 1 — Supplementary Information. [file 41598_2021_92869_MOESM1_ESM.pdf]

Supplemental Information

The maintenance of microbial community in human fecal samples by a cost effective preservation buffer

Chongming Wu[1,3,*], Tianda Chen[2,3], Wenyi Xu[2,3], Tingting Zhang[2], Yuwei Pei[2], Yanan Yang[1], Fang Zhang[1], Hao Guo[2], Qingshi Wang[2], Li Wang[2], Bowen Zhao[2,*]

[1]Pharmacology and Toxicology Research Center, Institute of Medicinal Plant Development, Chinese Academy of Medical Sciences & Peking Union Medical College, Beijing, 100193, China. [2]Beijing QuantiHealth Technology Co., Ltd., Beijing 100070, China. [3]These authors contributed equally: Chongming Wu, Tianda Chen and Wenyi Xu. [*] Emails: cmwu@implad.ac.cn and zhaobowen@quantibio.com

This PDF file includes:

Supplemental Figure S1. Clustering analysis of 9 samples under different temperatures.
Supplemental Figure S2. KEGG functional profiling of 9 samples under different temperatures.
Supplemental Table S1. Summary of reads number in Figure 1.
Supplemental Table S2. Summary of reads number in Figure 2-3.
Supplemental Table S3. Summary of reads number in Figure 4.

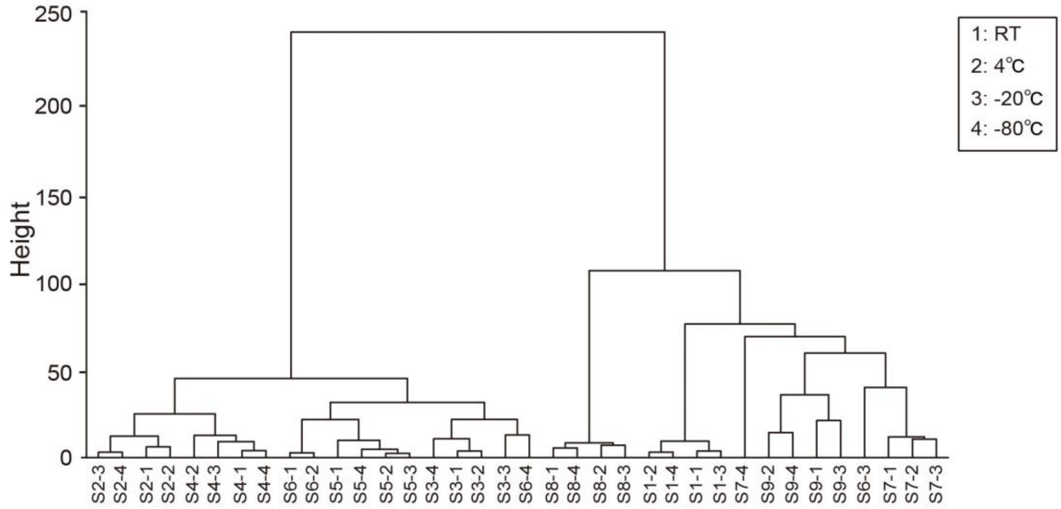

Supplemental Figure S1. Clustering analysis of 9 samples under different temperatures.

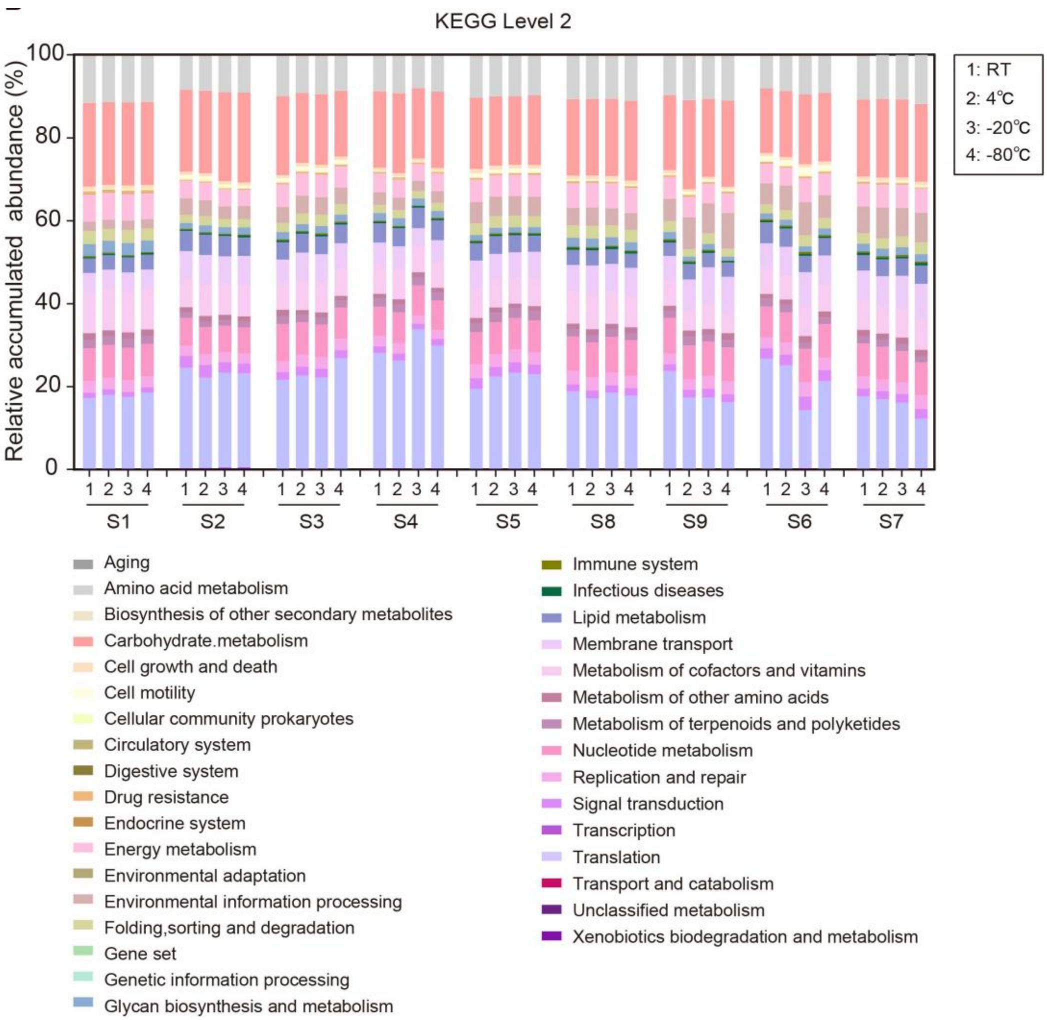

Supplemental Figure S2. KEGG functional profiling of 9 samples under different temperatures (https://www.kegg.jp/kegg/ko.html).

Supplemental Table S1. Summary of reads number in Figure 1.							
**Sample Type**	**Reads Number**	**Total Bases**	**Q20(%)**	**Q30(%)**	**Minimal Length**	**Maximal Length**	**Average Length**
**Note:** The capital letter Q indicates Quality Scores. Q20 represents the base call accuracy of 99%, and Q30 represents that of 99.9%, which is considered a benchmark for quality in next-generation.							
S1#1_RawData	17,332,698	2,599,904,700	97.25	94.25	150	150	150
S1#1_CleanData	16,158,524	1,873,264,490	100	99.06	30	147	115.9
S1#2_RawData	6,112,952	916,942,800	94.35	88.95	150	150	150
S1#2_CleanData	5,392,648	528,864,906	100	97.38	30	147	98.1
S1#3_RawData	11,401,938	1,710,290,700	96.67	93.26	150	150	150
S1#3_CleanData	10,677,092	1,218,734,632	100	98.89	30	147	114.1
S1#4_RawData	9,633,392	1,445,008,800	96.55	93.43	150	150	150
S1#4_CleanData	8,954,670	980,837,159	100	99.09	30	147	109.5
S2#1_RawData	19,681,650	2,952,247,500	96.62	92.75	150	150	150
S2#1_CleanData	17,498,292	2,092,219,250	100	98.82	30	146	119.6
S2#2_RawData	9,703,002	1,455,450,300	96.86	93.58	150	150	150
S2#2_CleanData	8,990,682	1,061,190,142	100	99.13	30	149	118
S2#3_RawData	11,736,488	1,760,473,200	97	93.65	150	150	150
S2#3_CleanData	10,812,332	1,249,912,851	100	99.12	30	148	115.6
S2#4_RawData	11,093,698	1,664,054,700	97.1	93.38	150	150	150
S2#4_CleanData	10,023,884	1,146,695,363	100	98.68	30	143	114.4
S3#1_RawData	58,922,870	8,838,430,500	97.2	93.84	150	150	150
S3#1_CleanData	54,013,228	6,483,969,904	100	98.93	30	147	120
S3#2_RawData	9,435,970	1,415,395,500	96.99	93.05	150	150	150
S3#2_CleanData	8,392,720	1,044,351,431	100	98.77	30	147	124.4
S3#3_RawData	23,318,554	3,497,783,100	97.19	93.52	150	150	150
S3#3_CleanData	20,984,898	2,592,696,959	100	98.83	30	147	123.6
S3#4_RawData	9,686,446	1,452,966,900	96.99	93.58	150	150	150
S3#4_CleanData	8,863,762	1,063,597,791	100	99.07	30	148	120
S4#1_RawData	7,978,444	1,196,766,600	97.14	93.65	150	150	150
S4#1_CleanData	7,430,192	878,614,921	100	98.81	30	146	118.2
S4#2_RawData	13,693,004	2,053,950,600	96.29	92.03	150	150	150
S4#2_CleanData	12,185,820	1,393,478,092	100	98.79	30	146	114.4
S4#3_RawData	10,978,658	1,646,798,700	96.54	92.59	150	150	150
S4#3_CleanData	10,056,914	1,167,801,424	100	98.71	30	147	116.1
S4#4_RawData	13,864,472	2,079,670,800	96.56	92.09	150	150	150
S4#4_CleanData	12,131,174	1,391,096,895	100	98.47	30	146	114.7
S5#1_RawData	10,767,436	1,615,115,400	96.79	92.51	150	150	150
S5#1_CleanData	9,537,982	1,147,998,369	100	98.72	30	147	120.4
S5#2_RawData	17,545,172	2,631,775,800	96.44	92.26	150	150	150
S5#2_CleanData	15,742,960	1,861,847,115	100	98.87	30	147	118.3
S5#3_RawData	7,976,898	1,196,534,700	97.01	93.11	150	150	150
S5#3_CleanData	7,137,764	861,303,813	100	98.85	30	146	120.7
S5#4_RawData	11,126,232	1,668,934,800	96.63	92.97	150	150	150
S5#4_CleanData	10,296,136	1,153,359,531	100	99.04	30	149	112
S6#1_RawData	7,675,496	1,151,324,400	95.65	92.32	150	150	150
S6#1_CleanData	7,196,098	744,866,729	100	99.22	30	149	103.5
S6#2_RawData	6,644,008	996,601,200	95.63	92.31	150	150	150
S6#2_CleanData	6,239,992	628,718,759	100	99.25	30	149	100.8
S6#3_RawData	11,015,186	1,652,277,900	95.28	91.96	150	150	150
S6#3_CleanData	10,415,516	981,737,661	100	99.29	30	149	94.3

S6#4_RawData	10,246,342	1,536,951,300	96.02	92.69	150	150	150
S6#4_CleanData	9,576,044	1,026,894,461	100	99.19	30	149	107.2
S7#1_RawData	8,168,416	1,225,262,400	95.23	91.24	150	150	150
S7#1_CleanData	7,547,662	746,686,990	100	99.1	30	149	98.9
S7#2_RawData	11,648,228	1,747,234,200	93.93	89.71	150	150	150
S7#2_CleanData	10,786,006	977,409,259	100	99.14	30	150	90.6
S7#3_RawData	6,791,638	1,018,745,700	91.21	86.02	150	150	150
S7#3_CleanData	6,018,380	488,745,166	100	99.16	30	149	81.2
S7#4_RawData	11,050,402	1,657,560,300	95.67	91.89	150	150	150
S7#4_CleanData	10,254,018	1,050,408,377	100	99.13	30	149	102.4
S8#1_RawData	13,617,668	2,042,650,200	95.48	91.63	150	150	150
S8#1_CleanData	12,611,104	1,303,454,753	100	99.11	30	149	103.4
S8#2_RawData	8,128,678	1,219,301,700	93.47	89.84	150	150	150
S8#2_CleanData	7,665,896	659,467,654	100	99.35	30	149	86
S8#3_RawData	7,927,990	1,189,198,500	92.65	86.79	150	150	150
S8#3_CleanData	7,011,514	609,696,033	100	97.67	30	149	87
S8#4_RawData	13,535,546	2,030,331,900	95.83	91.16	150	150	150
S8#4_CleanData	11,715,258	1,368,439,099	100	98.85	30	148	116.8
S9#1_RawData	8,492,470	1,273,870,500	96.51	93.33	150	150	150
S9#1_CleanData	7,975,956	865,961,299	100	99.19	30	149	108.6
S9#2_RawData	8,080,214	1,212,032,100	96.14	93.04	150	150	150
S9#2_CleanData	7,645,732	798,822,300	100	99.28	30	149	104.5
S9#3_RawData	6,236,566	935,484,900	91.96	87.9	150	150	150
S9#3_CleanData	5,866,544	452,382,786	100	99.37	30	150	77.1
S9#4_RawData	10,192,172	1,528,825,800	96.5	93.44	150	150	150
S9#4_CleanData	9,631,142	1,034,680,312	100	99.26	30	149	107.4

Supplemental Table S2. Summary of reads number in Figure 2-3.							
**Sample Type**	**Reads Number**	**Total Bases**	**Q20(%)**	**Q30(%)**	**Minimal Length**	**Maximal Length**	**Average Length**
S1#LN_RawData	14,857,370	2,228,605,500	98.45	94.87	150	150	150
S1#LN_CleanData	14,184,938	1,658,048,857	100	97.45	30	143	116.9
S1#AT-1d_RawData	11,361,232	1,704,184,800	96.92	91.9	150	150	150
S1#AT-1d_CleanData	10,032,094	1,035,814,059	100	96.68	30	147	103.3
S1#AT-3d_RawData	10,773,230	1,615,984,500	96.89	91.6	150	150	150
S1#AT-3d_CleanData	9,362,618	923,195,877	100	96.44	30	143	98.6
S1#AT-1W_RawData	20,213,124	3,031,968,600	98.43	94.88	150	150	150
S1#AT-1W_CleanData	19,291,472	2,254,005,468	100	97.47	30	143	116.8
S1#AT-2W_RawData	12,927,472	1,939,120,800	97.79	94.33	150	150	150
S1#AT-2W_CleanData	12,251,860	1,435,280,452	100	97.78	30	146	117.1
S1#AT-4W_RawData	16,520,472	2,478,070,800	98.23	94.64	150	150	150
S1#AT-4W_CleanData	15,714,168	1,839,443,015	100	97.52	30	144	117.1
S1#PB-AT-1d_RawData	7,519,866	1,127,979,900	96.76	92.06	150	150	150
S1#PB-AT-1d_CleanData	6,903,528	694,016,521	100	97.17	30	147	100.5
S1#PB-AT-3d_RawData	7,631,754	1,144,763,100	96.2	91.6	150	150	150
S1#PB-AT-3d_CleanData	7,049,090	698,655,992	100	97.32	30	148	99.1
S1#PB-AT-1W_RawData	15,453,016	2,317,952,400	97.69	93.64	150	150	150
S1#PB-AT-1W_CleanData	14,535,094	1,599,131,499	100	97.57	30	147	110
S1#PB-AT-2W_RawData	13,899,828	2,084,974,200	98.1	94.29	150	150	150
S1#PB-AT-2W_CleanData	13,090,156	1,481,038,551	100	97.45	30	143	113.1
S1#PB-AT-4W_RawData	10,541,468	1,581,220,200	96.18	91.57	150	150	150
S1#PB-AT-4W_CleanData	9,763,938	1,022,578,503	100	97.33	30	148	104.7
S1#PB(2W)-HT-3d_RawData	9,900,348	1,485,052,200	96.84	92.54	150	150	150
S1#PB(2W)-HT-3d_CleanData	9,235,648	990,086,890	100	97.4	30	148	107.2
S1#PB(2W)-HT-4d_RawData	11,655,366	1,748,304,900	97.48	93.14	150	150	150
S1#PB(2W)-HT-4d_CleanData	10,880,572	1,198,664,479	100	97.23	30	147	110.2
S1#PB(2W)-HT-5d_RawData	9,779,502	1,466,925,300	96.52	92.03	150	150	150
S1#PB(2W)-HT-5d_CleanData	9,098,482	960,217,487	100	97.36	30	148	105.5
S2#LN_RawData	13,787,078	2,068,061,700	97.31	92.88	150	150	150
S2#LN_CleanData	12,771,716	1,380,736,329	100	97.25	30	147	108.1
S2#AT-1d_RawData	7,740,656	1,161,098,400	94.2	88.77	150	150	150
S2#AT-1d_CleanData	6,887,134	600,159,119	100	97.34	30	149	87.1
S2#AT-3d_RawData	7,613,212	1,141,981,800	94.06	88.39	150	150	150
S2#AT-3d_CleanData	6,797,792	590,508,153	100	97.2	30	149	86.9
S2#AT-1W_RawData	9,639,120	1,445,868,000	98.33	94.96	150	150	150
S2#AT-1W_CleanData	9,315,814	1,078,635,306	100	97.67	30	148	115.8
S2#AT-2W_RawData	14,940,328	2,241,049,200	97.28	93.08	150	150	150
S2#AT-2W_CleanData	13,984,084	1,523,230,930	100	97.48	30	148	108.9
S2#AT-4W_RawData	6,101,268	915,190,200	98.56	95.22	150	150	150
S2#AT-4W_CleanData	5,894,088	686,756,957	100	97.6	30	146	116.5
S2#PB-AT-1d_RawData	8,874,596	1,331,189,400	97.55	93.24	150	150	150
S2#PB-AT-1d_CleanData	8,237,544	867,377,072	100	97.08	30	146	105.3
S2#PB-AT-3d_RawData	10,060,102	1,509,015,300	97.27	92.76	150	150	150
S2#PB-AT-3d_CleanData	9,224,994	942,162,067	100	96.97	30	144	102.1
S2#PB-AT-1W_RawData	13,662,212	2,049,331,800	97.69	93.6	150	150	150
S2#PB-AT-1W_CleanData	12,747,428	1,409,460,646	100	97.41	30	146	110.6
S2#PB-AT-2W_RawData	14,201,766	2,130,264,900	97.63	93.65	150	150	150
S2#PB-AT-2W_CleanData	13,380,866	1,494,757,836	100	97.51	30	147	111.7
S2#PB-AT-4W_RawData	15,294,214	2,294,132,100	97.49	93.38	150	150	150
S2#PB-AT-4W_CleanData	14,313,248	1,584,653,306	100	97.48	30	148	110.7

S2#PB(2W)-HT-3d_RawData	15,459,692	2,318,953,800	97.38	93.08	150	150	150
S2#PB(2W)-HT-3d_CleanData	14,396,008	1,557,879,224	100	97.33	30	148	108.2
S2#PB(2W)-HT-4d_RawData	11,688,826	1,753,323,900	97.77	93.8	150	150	150
S2#PB(2W)-HT-4d_CleanData	11,003,428	1,223,101,884	100	97.59	30	147	111.2
S2#PB(2W)-HT-5d_RawData	11,965,560	1,794,834,000	97.58	93.57	150	150	150
S2#PB(2W)-HT-5d_CleanData	11,258,026	1,248,810,126	100	97.53	30	147	110.9
S3#LN_RawData	10,542,986	1,581,447,900	97.18	92.91	150	150	150
S3#LN_CleanData	9,786,854	1,036,332,055	100	97.63	30	147	105.9
S3#AT-1d_RawData	14,915,526	2,237,328,900	97.29	92.69	150	150	150
S3#AT-1d_CleanData	13,556,018	1,391,751,307	100	96.85	30	143	102.7
S3#AT-3d_RawData	6,759,830	1,013,974,500	94.81	89.49	150	150	150
S3#AT-3d_CleanData	6,075,242	565,786,079	100	97.22	30	149	93.1
S3#AT-1W_RawData	8,889,094	1,333,364,100	95.1	90.32	150	150	150
S3#AT-1W_CleanData	8,143,310	809,642,477	100	97.62	30	149	99.4
S3#AT-2W_RawData	14,888,728	2,233,309,200	97.4	93.15	150	150	150
S3#AT-2W_CleanData	13,682,892	1,521,823,653	100	97.34	30	144	111.2
S3#AT-4W_RawData	17,701,162	2,655,174,300	97.71	93.49	150	150	150
S3#AT-4W_CleanData	16,329,106	1,830,521,117	100	97.25	30	144	112.1
S3#PB-AT-1d_RawData	9,248,050	1,387,207,500	97.35	93.05	150	150	150
S3#PB-AT-1d_CleanData	8,583,412	900,001,552	100	97.16	30	146	104.9
S3#PB-AT-3d_RawData	7,930,174	1,189,526,100	96.91	92.5	150	150	150
S3#PB-AT-3d_CleanData	7,347,152	754,715,510	100	97.24	30	149	102.7
S3#PB-AT-1W_RawData	15,353,930	2,303,089,500	96.91	91.76	150	150	150
S3#PB-AT-1W_CleanData	13,569,308	1,415,244,829	100	96.77	30	143	104.3
S3#PB-AT-2W_RawData	13,757,186	2,063,577,900	97.74	93.79	150	150	150
S3#PB-AT-2W_CleanData	12,819,410	1,480,140,307	100	97.51	30	147	115.5
S3#PB-AT-4W_RawData	12,907,900	1,936,185,000	96.19	91.66	150	150	150
S3#PB-AT-4W_CleanData	11,838,380	1,269,914,417	100	97.42	30	148	107.3
S3#PB(2W)-HT-3d_RawData	17,751,686	2,662,752,900	97.81	93.79	150	150	150
S3#PB(2W)-HT-3d_CleanData	16,389,310	1,862,016,879	100	97.42	30	143	113.6
S3#PB(2W)-HT-4d_RawData	13,632,330	2,044,849,500	97.72	93.84	150	150	150
S3#PB(2W)-HT-4d_CleanData	12,781,598	1,462,395,279	100	97.59	30	147	114.4
S3#PB(2W)-HT-5d_RawData	14,419,018	2,162,852,700	97.87	93.92	150	150	150
S3#PB(2W)-HT-5d_CleanData	13,441,920	1,516,984,777	100	97.45	30	143	112.9

Supplemental Table S3. Summary of reads number in Figure 4.

Sample Type	Reads Number	Total Bases	Q20(%)	Q30(%)	Minimal Length	Maximal Length	Average Length
S1#-80 ℃ RawData	18,456,254	2,768,438,100	95.86	90.77	150	150	150
S1#-80 ℃ CleanData	1,913,990	205,120,557	100	97.91	30	149	107.2
S1#PB_RawData	20,092,332	3,013,849,800	96.47	92.12	150	150	150
S1#PB_CleanData	4,208,276	481,179,144	100	98.35	30	143	114.3
S2#-80 ℃ RawData	16,786,482	2,517,972,300	95.7	90.39	150	150	150
S2#-80 ℃ CleanData	3,559,106	396,771,920	100	97.99	30	149	111.5
S2#PB_RawData	18,694,410	2,804,161,500	94.73	88.78	150	150	150
S2#PB_CleanData	5,189,462	571,700,729	100	97.92	30	143	110.2
S3#-80 ℃ RawData	17,716,730	2,657,509,500	96.34	91.99	150	150	150
S3#-80 ℃ CleanData	1,189,510	133,471,897	100	98.27	30	149	112.2
S3#PB_RawData	14,347,950	2,152,192,500	97.91	95.37	150	150	150
S3#PB_CleanData	1,530,522	191,454,622	100	99.04	30	140	125.1
S4#-80 ℃ RawData	15,175,150	2,276,272,500	96.29	91.55	150	150	150
S4#-80 ℃ CleanData	4,583,158	523,932,551	100	98.21	30	149	114.3
S4#PB_RawData	16,820,682	2,523,102,300	96.39	92.43	150	150	150
S4#PB_CleanData	6,214,306	769,854,617	100	98.79	30	143	123.9
S5#-80 ℃ RawData	12,201,130	1,830,169,500	97.37	94.11	150	150	150
S5#-80 ℃ CleanData	587,074	66,313,695	100	98.49	30	149	113
S5#PB_RawData	15,901,400	2,385,210,000	97.39	94.4	150	150	150
S5#PB_CleanData	650,840	80,350,605	100	98.7	30	149	123.5
